# Acute Superoxide Radical Scavenging Reduces Blood Pressure but Does Not Influence Kidney Function in Hypertensive Rats with Postischemic Kidney Injury

**DOI:** 10.1155/2014/512619

**Published:** 2014-06-22

**Authors:** Zoran Miloradović, Milan Ivanov, Nevena Mihailović-Stanojević, Jelica Grujić Milanović, Đurđica Jovović, Una-Jovana Vajić, Jasmina Marković-Lipkovski

**Affiliations:** ^1^Department of Cardiovascular Physiology, Institute for Medical Research, University of Belgrade, 11129 Belgrade, Serbia; ^2^Institute of Pathology, Medical School, University of Belgrade, 11129 Belgrade, Serbia

## Abstract

Acute kidney injury (AKI) is associated with significant morbidity and mortality in hypertensive surroundings. We investigated superoxide radical molecules influence on systemic haemodynamic and kidney function in spontaneously hypertensive rats (SHR) with induced postischemic AKI. Experiment was performed in anesthetized adult male SHR. The right kidney was removed, and left renal artery was subjected to ischemia by clamping for 40 minutes. The treated group received synthetic superoxide dismutase mimetic TEMPOL in the femoral vein 5 minutes before, during, and 175 minutes after the period of reperfusion, while the control AKI group received the vehicle via the same route. All parameters were measured 24 h after renal reperfusion. TEMPOL treatment significantly decreased mean arterial pressure and total peripheral resistance (*P* < 0.05) compared to AKI control. It also increased cardiac output and catalase activity (*P* < 0.05). Lipid peroxidation and renal vascular resistance were decreased in TEMPOL (*P* < 0.05). Plasma creatinine and kidney morphological parameters were unchanged among TEMPOL treated and control groups. Our study shows that superoxide radicals participate in haemodynamic control, but acute superoxide scavenging is ineffective in glomerular and tubular improvement, probably due to hypertension-induced strong endothelial dysfunction which neutralizes beneficial effects of O_2_^−^ scavenging.

## 1. Introduction

Acute kidney injury (AKI), commonly caused by an obstruction of renal blood flow (renal ischemia) and characterized by a rapid decline in glomerular filtration rate (GFR) [1], is frequently associated with significant morbidity and mortality, the latter in the range of 30–60%, depending on the clinical setting and presence or absence of nonrenal organ failure [2]. Unfortunately, therapeutic approaches to prevent or treat ARF are extremely limited, as the majority of interventional trials in AKI have failed in humans [3, 4]. Therefore, the search for novel therapeutic modalities to prevent or treat AKI represents an area of intense investigation.

There are several factors involved in the initiation and maintenance of the AKI: decrease of glomerular capillary permeability, backleak of glomerular filtrate, tubular obstruction, and intrarenal vasoconstriction [1], but their causality has never been selective or rarely preventable. On the other hand, long-lasting hypertension damages medium-size and small-size renal blood vessels, disables adequate tubuloglomerular responses, and predisposes nephroangiosclerosis patients to AKI [5]. Also, patients with preexisting hypertension are at a particular risk of fatal outcome during AKI [6, 7].

Oxidative stress appears as a main mechanism causing tissue ischemia-reperfusion injury. Reperfusion injury generates a significant amount of free reactive oxygen species (ROS), the effect of which could not be “buffered” by endothelial cells exposed to ischemia [8]. Many studies have shown that ROS could induce lipid peroxidation, increase plasma membrane permeability [9], modulate both enzyme and membrane pump activity [10], and induce damage of DNA molecule [11, 12]. Study of Paller [13] has shown that antioxidants, including allopurinol, exert protective effects on postischemic kidney in rats, dogs, mice, rabbits, and pigs. On the other hand, study of Scaduto et al. [14] showed that allopurinol is ineffective in ischemic model of AKI. There are controversies related to efficacy of other antioxidative molecules in ischemic injury. Glutathione, normally present in tubulocites, expresses antioxidative properties, but its concentration markedly drops after renal ischemia [15]. Besides, glutathione therapy has shown opposite effects in ischemia-reperfusion injury [16, 17].

Therefore, in the present study, by using oxidative scavenging therapy, we sought to determine whether superoxide radical molecules influence haemodynamic, lipid peroxidation and kidney function in spontaneously hypertensive rats (SHR) with induced postischemic AKI.

## 2. Materials and Methods

Male adults SHR, 24 weeks old, weighing about 300 g, were bred in the Institute for Medical Research, Belgrade, Serbia, and fed with a standard chow for laboratory rats (Veterinarski Zavod, Subotica, Serbia).

All animal experiments were conducted in accordance with local institutional guidelines for the care and use of laboratory animals. The investigation also conformed to the principles and guidelines of Conseil de l'Europe (published in the Official Daily N. L358/1-358/6, 18 December, 1986), the US National Institutes of Health (Guide for the Care and Use of Laboratory Animals, NIH publication number 85-23), and the Canadian Council on Animal Care (CCAC).

### 2.1. Experimental Protocol

All experiments were performed in anaesthetized (35 mg/kg b.m. sodium pentobarbital; intraperitoneal (i.p.)) rats.

### 2.2. Experimental Groups and Design

Hypertension was confirmed in all rats by indirect measurement on tail artery (Narco Bio Systems Inc., Houston, TX, USA), and the animals were divided into the following experimental groups: sham operated rats (SHAM); rats with induced postischemic AKI (AKI control); group AKI + TEMPOL, which received superoxide dismutase (SOD) mimetic TEMPOL (4-hydroxy-2,2, 6,6-tetramethylpiperidine-1-^15^N-oxyl; Sigma Chemical Co., USA) during ARF induction.

AKI was induced by surgical removal of right kidney followed by atraumatic clamp occlusion of the left renal artery for 40 minutes. Control rats received vehicle (saline), while AKI + TEMPOL group received SOD mimetic TEMPOL (40 mg/kg/h b.w.), in the femoral vein 5 minutes before and 175 minutes after the clamp removal. After infusion, the wound abdominal incision was surgically closed and SHR were placed individually into metabolic cages for 24 hours, with free access to water and chow.

### 2.3. Haemodynamic Measurements 24 Hours after Reperfusion

Haemodynamic parameters were measured in anaesthetized rats, through a femoral artery catheter (PE-50, Clay-Adams Parsippany, NY, USA), connected to a physiological data acquisition system (9800TCR Cardiomax III-TCR, Columbus, OH, USA). A jugular vein was cannulated with polyethylene tubing PE-50 for the injection of cold saline. The left carotid artery was catheterized with PE-50 tubing and attached to a Thermo Sensor, which was coupled to the Cardiomax III for the determination of cardiac output (CO). The other end of thermocouple was placed in cold saline. Following 20 min for stabilization after surgery, cold saline (0.2 mL) was supplied through the jugular vein and mean arterial pressure (MAP), heart rate (HR), and CO were recorded. Total peripheral vascular resistance (TPVR) was calculated from MAP and CO (assuming that mean right atrial pressure is zero) and expressed as mmHg min kg/mL.

After abdominal incision, left renal artery preparation was utilized and an ultrasonic flow probe (1RB, internal diameter = 1 mm) was placed around the artery for the measurement of renal blood flow (RBF), using a Transonic T106 Small Animal Flowmeter (Transonic System Inc., Ithaca, NY, USA). Vascular resistance in renal artery (RVR) was calculated by dividing MAP by RBF, normalized for the body weight and expressed as mmHg × min × kg/mL.

### 2.4. Biochemical Measurements 24 h after Reperfusion

After haemodynamic studies, blood samples were taken for determination of creatinine (P_Cr_) and urea (P_U_) in plasma. Lithium-heparin (Li-heparin, Sigma, USA) was used as an anticoagulant. 24 h urine samples were used for determination of urine creatinine (U_Cr_) and urea (U_U_) concentrations in urine. All biochemical parameters were measured using an automatic COBAS INTEGRA 400 plus (Hoffmann-La Roche, Germany) analyzer. Creatinine (C_Cr_) and urea (C_U_) clearances were calculated according to standard formula and normalized to body weight. After blood samples collection, animals were sacrificed by pentobarbital overdose injection.

Further, blood samples were centrifuged at 3000 rpm at 4°C for 15 minutes and erythrocytes were separated. Hemoglobin (Hb) content was estimated by the method of Drabkin and Austin [17]. Spectrophotometric analyses of plasma or erythrocytes were performed in Ultrospec 3300 pro UV/Visible spectrophotometer, Amersham Biosciences Corp., USA.

Activity of catalase (CAT) in erythrocytes was determined according to the procedure of Beutler [18] by following the absorbance of hydrogen peroxide at 230 nm. The activity of this enzyme was expressed as unit per gram of hemoglobin (U/g Hg) where one unit of CAT activity was defined as mmol of H_2_O_2 per_ minute per gram of hemoglobin.

Thiobarbituric acid reactive substances (TBARS), as a marker of lipid peroxidation, were measured by using 2-thiobarbituric acid (2,6-dihydroxypyrimidine-2-thiol; TBA). An extinction coefficient of 156000 M-1 cm-1 was used for calculation [19] and level of TBARS was expressed as nmol per milliliter of plasma.

### 2.5. Histological Examination

For determination of morphological changes, the left kidney was removed immediately after sacrificing and than prepared for light microscopy. The renal tissue was fixed in 10% buffered formalin solution. Later, the kidney was dehydrated in alcohol and blocked in paraffin wax, and 5 *μ*m thick sections were sliced and stained by periodic acid-Schiff (PAS) reaction. By light microscopy the following parameters were semiquantitatively evaluated on the scale from 0 to 4 according to the degree of lesions: intensity and spread of tubular necrosis, number of intraluminal cast formations, swelling and vacuolization of cells, loss of luminal membrane or brush borders, tubular dilatation, interstitial oedema, and separation of cells from tubular basal membrane. The severity of congestion, that is, the accumulation of red blood cells in glomeruli, peritubular capillaries, and intrarenal veins, was graded on a scale from 1 to 3, as described by Mandal et al. [20]. The sum of these changes represented the histopathological score for comparison between groups. Two independent investigators made histological evaluations; consensus was reached by discussion, whereas for the pathohistological score the mean value was calculated for each group.

### 2.6. Statistical Analysis

The results are expressed as mean ± SEM. For results processing, we used the single-sided Student's *t*-test for two samples of equal variance (Microsoft Excel 2010). This test was used for comparing AKI control and SHAM, as well as AKI + TEMPOL and AKI control groups. *P* values < 0.05 were considered significant.

## 3. Results

### 3.1. Haemodynamic Measurements

**Figure 1:**
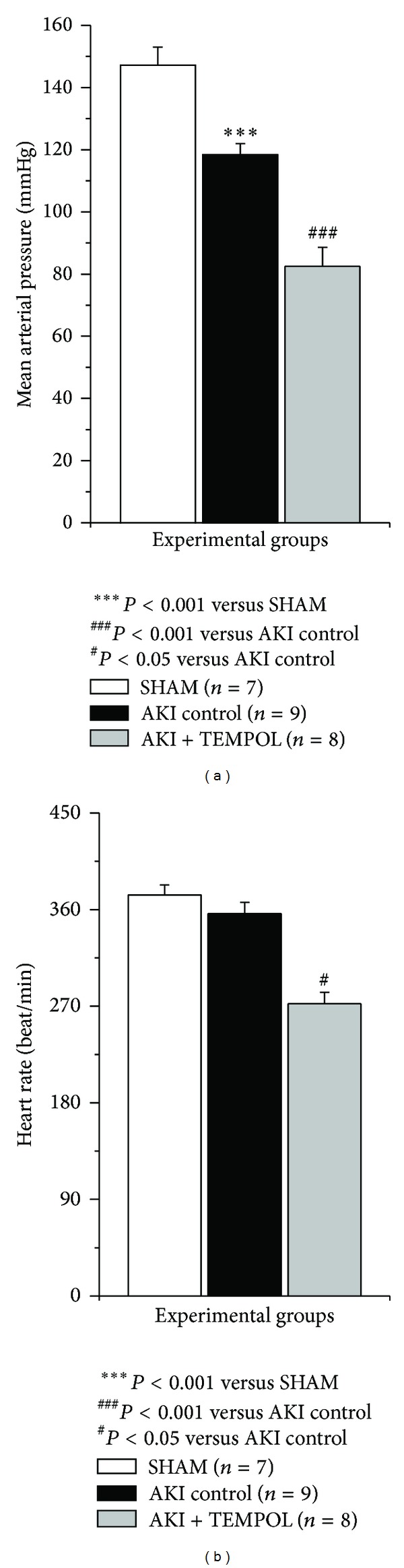
Mean arterial pressure and heart rate in experimental group 24 hours after reperfusion.

**Figure 2:**
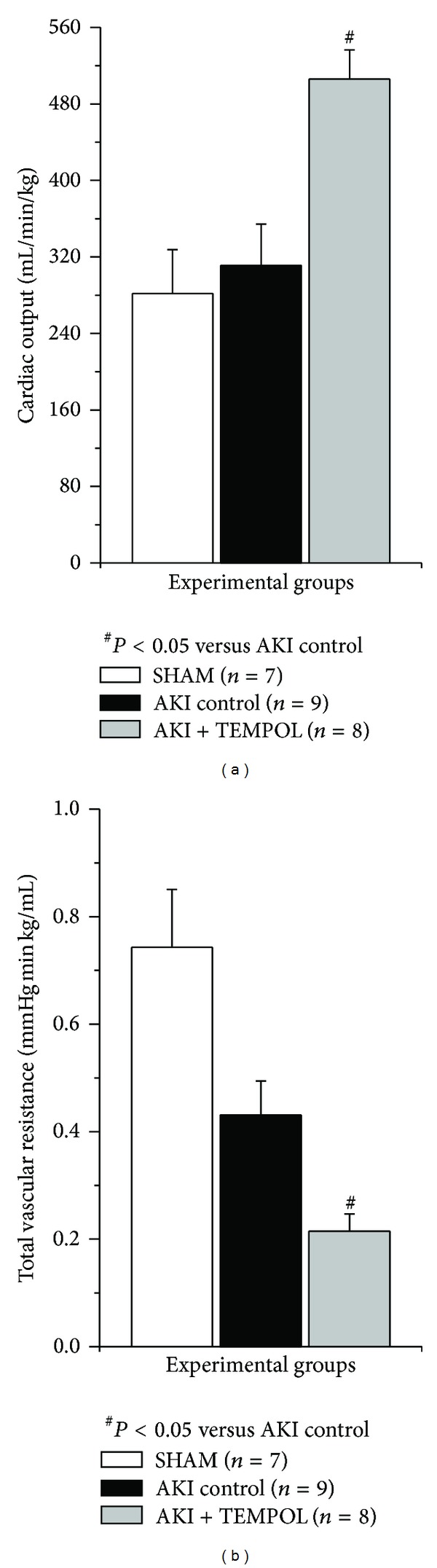
Cardiac output and total vascular resistance in experimental group 24 hours after reperfusion.

Both MAP and HR were reduced in TEMPOL treated animals in comparison to AKI control (*P* < 0.001 and *P* < 0.05; Figure 1). Furthermore, TPVR was significantly decreased (*P* < 0.05) and CO was significantly increased in SOD mimetic treated group versus control (*P* < 0.05) (Figure 2).

**Figure 3:**
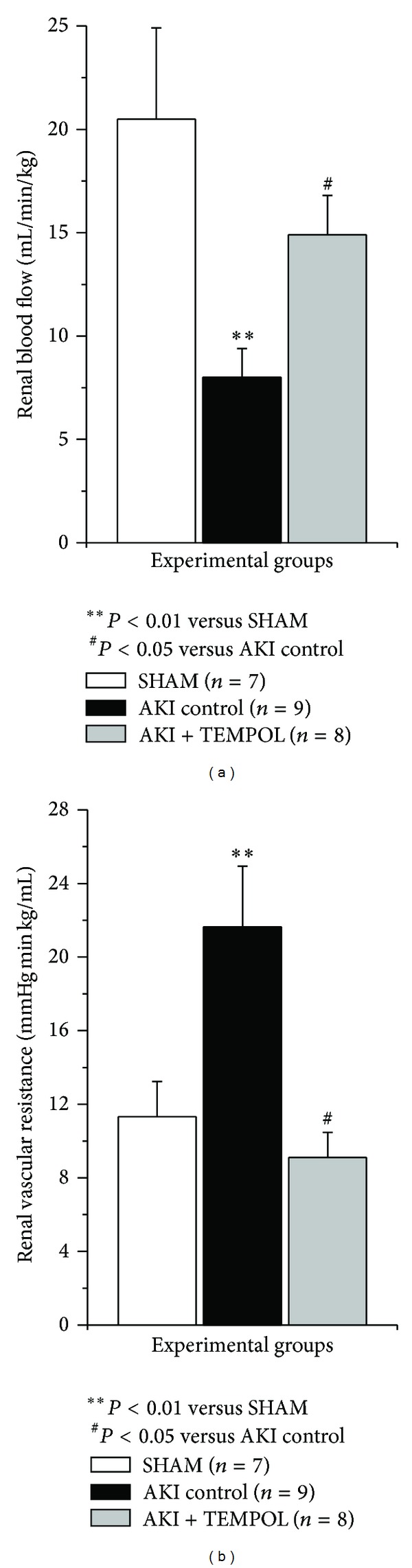
Renal blood flow and renal vascular resistance in experimental group 24 hours after reperfusion.

AKI induction significantly reduced RBF and increased RVR in comparison to sham operated animals. TEMPOL infusion significantly increased RBF and reduced RVR (*P* < 0.05) in AKI + TEMPOL group in comparison to AKI control (Figure 3).

### 3.2. Biochemical Parameters

After AKI induction plasma creatinine was significantly increased in AKI control group as compared to sham operated animals (*P* < 0.001). TEMPOL treatment had no influence on this glomerular filtration marker. Also, TEMPOL infusion changed neither Pu nor Pphos levels in postischemic SHR (Table 1). Similar relation was observed in both clearances. AKI induction significantly reduced C_Cr_ and C_U_, while TEMPOL treatment had no influence on glomerular filtration improvement (Table 1).

Enzyme catalase activity dropped after AKI induction, while TEMPOL treatment significantly elevated its activity (*P* < 0.05) 24 hours after ischemia (Table 1).

TBARS level was significantly increased in AKI group. TEMPOL treatment significantly reduced lipid peroxidation (*P* < 0.01) (Table 1).

### 3.3. Histological Studies

**Figure 4:**
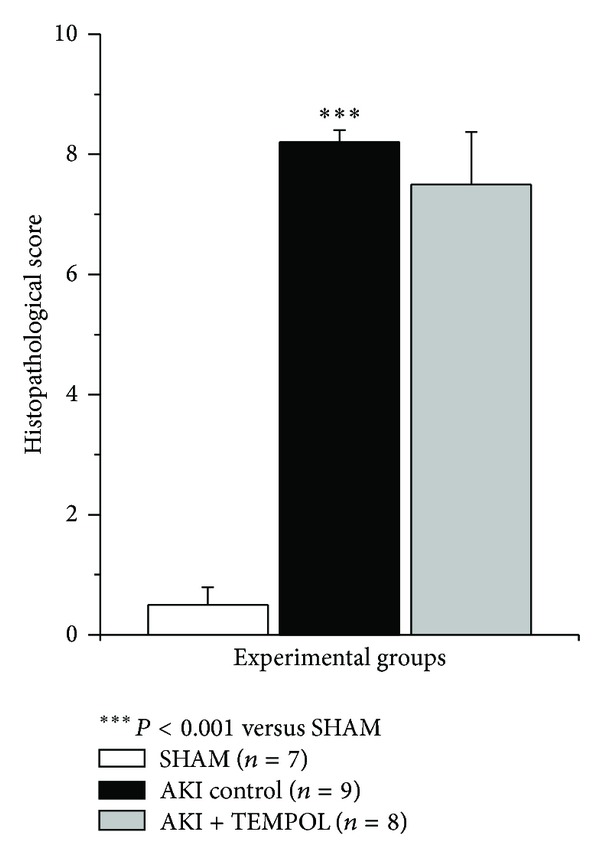
Histopathological score in experimental group 24 hours after reperfusion.

Histological examination of the kidney specimens obtained 24 hours after AKI revealed that rats treated with TEMPOL had minor morphological changes in comparison to AKI control group (Figure 4).

**Figure 5:**
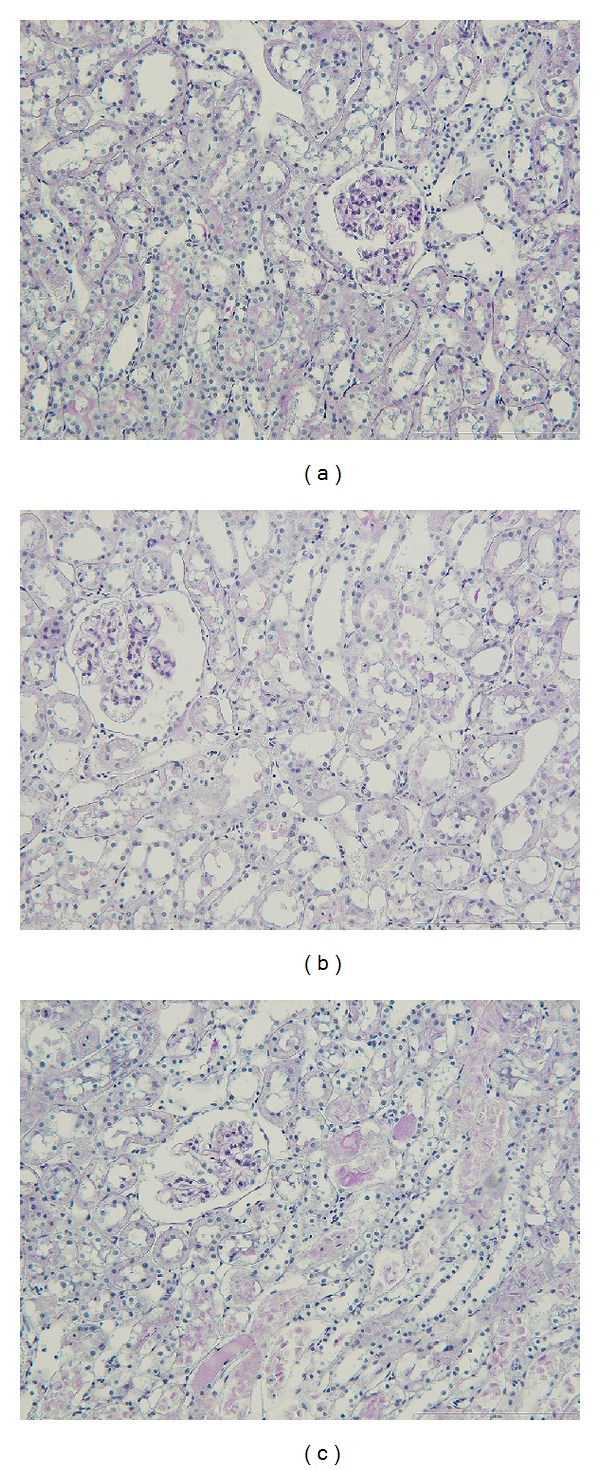
(a) Normal appearance of glomeruli, interstitium, tubules, and blood vessels in SHAM operated animals. Very rare PAS positive casts in the lumen of the tubules. (b) Massive corticomedullary tubular necrosis (solid arrow). Intensive interstitial edema. Numerous PAS positive casts in the collecting ducts and dilatation of certain segments (dash arrow) of the proximal and distal tubules (with or without loss of brush border) in AKI control group. (c) AKI + TEMPOL group. Noticeable tubular dilatation. Slightly reduced tubular necrosis in the corticomedullary zone, with interstitial edema. Tubular casts in the renal medulla is comparable to AKI control animals.

Figure 5(a) shows the normal appearance of glomeruli, interstitium, tubules, and blood vessels in SHAM operated animals. Only in a few kidney specimens were observed a less number of PAS positive casts in the tubular lumen.

The kidneys of animals with AKI showed dilatation of certain segments of the proximal and distal tubules, with or without loss of brush border. The most notable changes were present in the corticomedullary zone where the broad areas of necrosis of tubules and a large number of PAS positive casts in the collecting ducts were observed. The intensity of interstitial edema in this group varies from sample to sample (Figure 5(b)).

In TEMPOL treated animals, similar damage is noticed in comparison to kidneys of control AKI animals. Tubular dilatation is noticeable. In the corticomedullary zone, tubular necrosis is slightly reduced and infarct fields have similar intensity as AKI control animals. Interstitial edema is observed. In addition, the number of tubular casts in the renal medulla is comparable to AKI control animals (Figure 5(c)).

## 4. Discussion 

In this study we examined the role of ROS in experimental genetically induced hypertension during postischemic AKI development. After AKI induction we noticed both mild MAP and HR reduction, similar to the results of Bowmer et al. [21] performed in the model of glycerol induced AKI. These authors considered high uremia (plasma urea was significantly elevated in AKI group in our study as well) influence on autonomic nervous system (diminished *α*_1_ adrenoreceptors sensitivity) as a cause of both MAP and HR reduction after AKI. Many studies have shown that superoxide radical scavenging reduces blood pressure in SHR [22–24]. Nishiyama et al. [25] have shown reduction of both blood pressure and TPVR due to O_2_^−^ scavenging in Ang II induced hypertension. In our experimental setting, TEMPOL treatment resulted in blood pressure lowering, followed by CO increasing and concomitant TPVR reduction in comparison to control AKI animals. Considering these results, it is reasonable to conclude that O_2_^−^ generation affects blood pressure regulation in the presented postischemic kidney hypertensive model.

Renal vasoconstriction is one of the major pathogenetic mechanisms of AKI development; therefore control of the renal haemodynamic is essential for this disorder. Schnackenberg et al. [22] have shown that TEMPOL (12.4 mg/kg t.m.) significantly reduced RVR without influencing RBF in SHR. Also, study of de Richelieu [26] showed chronic treatment with TEMPOL (1 mM, 15 days) towards effective RVR reduction in SHR. Furthermore, Li et al. [27] suggest that TEMPOL normalizes the RBF in SHR. Results of our study are in accordance with these studies, indicating that ROS scavenging is beneficial in reduction of postischemic induced renal vasoconstriction in SHR. Majid and Kopkan [28] showed that TEMPOL increased blood flow after angiotensin II induced oxidative stress in hypertensive rats. It is well known that postischemic AKI induces massive production of O_2_^−^ radicals [29], which have important role in human and animal hypertension [30–35]. Besides, there is increased production of NADPH oxidase iRNA in renal tissue of SHR, which is responsible for O_2_^−^ generation [35]. Cai and Harrison showed [36] that overproduction of O_2_^−^ is closely related to endothelial dysfunction and vasoconstriction. Therefore, we can conclude that, in our experimental model, both blood pressure lowering and renal artery vasodilatation in TEMPOL treated animals are mainly due to O_2_^−^ scavenging.

In the present study, there was a sevenfold increase of both plasma creatinine and urea and otherwise dramatically dropping of creatinine and urea clearance after AKI induction. The former indicates detrimental glomerular filtration after ischemia-reperfusion injury. TEMPOL treatment did not affect these parameters of kidney function. Chatterjee [37] reported plasma creatinine lowering due to TEMPOL treatment after kidney injury in* Wistar* rats. By our opinion, discrepancies among these studies could be explained by hypertensive postischemic milieu in SHR, which causes strong renal endothelial dysfunction and therefore abolishes positive effects of O_2_^−^ scavenging 24 hours after reperfusion. Hyperphosphatemia frequently occurs after AKI, due to diminished expression of tubular sodium dependent phosphate cotransporter [38]. In our experimental model, TEMPOL treatment did not change phosphatemia, indicating O_2_^−^ scavenging ineffective in tubular injury amelioration. This observation was confirmed by morphologically unchanged tubular structure in AKI + TEMPOL group, in comparison to AKI control. There is evidence that ischemia-reperfusion injury results in decreased erythrocyte catalase activity [39], what is also noticed in our experimental study. Hence, TEMPOL treatment induced twofold increasing of CAT activity; this should diminish oxidative stress during postischemic AKI development in our study. Besides, Yuan et al. [40] showed CAT activity positively correlated with elevated antioxidant superoxide dismutase enzyme. Another study [41] showed CAT mimetic property of TEMPOL. On the other hand, lipid peroxidation is closely related to cell structure damage. There were many studies showing elevated lipid peroxidation in plasma, erythrocytes, and kidney tissue, after renal ischemia [13, 42, 43]. TEMPOL treatment in our hypertensive rats decreased plasma lipid peroxidation which correlates with results of Zhang et al. [44]. They showed TEMPOL (10 mg/kg and 30 mg/kg b.w.) effective in TBARS lowering at Sprague-Dawley Ang II hypertensive rats. Considering that NAD(P)H oxidase is one of the main sources generating ROS [45] and that superoxide scavenging in our study resulted in both CAT activity increasing and lipid peroxidation decreasing, we could be free to conclude that TEMPOL treatment probably diminished activity of NADPH oxidase as well. Our results show that morphological changes in the kidney of TEMPOL treated animals were similar to control AKI rats. Although some data [37] showed TEMPOL treatment successful against postischemic kidney injury in normotensive conditions, our results indicate that hypertensive milieu in the kidneys of SHR suppresses morphological renal recovery after superoxide radical scavenging.

Taken together, results of our study show that acute TEMPOL treatment can diminish hypertension and improve systemic haemodynamic parameters after postischemic renal injury, suggesting that postischemic induced superoxide anions at least in one part participate in blood pressure and haemodynamic control in this condition. On the other hand, presented results indicate that acute superoxide scavenging is ineffective in general kidney function improvement, despite renal haemodynamic improvement 24 hours after reperfusion. In our opinion, this is due to strong renal endothelial dysfunction induced by hypertension, which neutralizes beneficial effect of acute O_2_^−^ scavenging. Thus, tubular injuries as main targets of postischemic episode remain mostly unaffected. Beyond this disappointment, there is a fact that TEMPOL treatment did not additionally decline glomerular function in postischemic SHR, so it could be effective in blood pressure therapy in hypertensive AKI patients. On the other hand, there are many open questions related to efficacy of long lasting TEMPOL treatment after postischemic kidney injury in hypertension or to protective influence of superoxide scavenging in recovery phase of AKI which could be topic for more complex studies.

## Figures and Tables

**Table 1:** Biochemical parameters in experimental groups 24 hours after reperfusion.

	Plasma creatinine (*µ*mol/L)	Plasma urea (mmol/L)	Creatinine clearance (mL/min/kg)	Urea clearance (mL/min/kg)	Plasma phosphates (mmol/L)	Catalase activity (U/g Hb)	TBARS (nmol/mL)
SHAM (*n* = 7)	32.71 ± 3.94	12.37 ± 1.70	6.50 ± 0.99	2.38 ± 0.28	2.39 ± 0.39	22.15 ± 6.05	7.28 ± 0.76
AKI control (*n* = 9)	242.71 ± 20.24***	61.90 ± 3.93***	0.29 ± 0.13***	0.11 ± 0.04***	5.57 ± 0.61***	14.32 ± 2.91	10.54 ± 0.92**
AKI + TEMPOL (*n* = 8)	225.63 ± 22	57.36 ± 3.17	0.32 ± 0.06	0.11 ± 0.02	4.25 ± 0.60	27.57 ± 6.36^#^	7.24 ± 0.37^##^

**P < 0.01 compared to SHAM; ***P < 0.001 compared to SHAM; ^#^ *P* < 0.05 compared to AKI control; ^##^ *P* < 0.01 compared to AKI control.
